# Remarks on Croup

**Published:** 1839-02-09

**Authors:** C. P. P. F. Reiersen


### REMARKS ON CROUP.

### By C. P. P. F. Reiersen, M. D.

To the Editors of the Medical Examiner.

*Gentlemen:*—The attention of the medical profession has of late been particularly directed to this disease, by the recent publication of the works of W. H. Porter, Frederic Ryland, Williams and others. The readers of the Examiner have lately had presented to them the fruits of the long experience of an able observer, Dr. C. D. Meigs. From the difference of opinion which prevails as to this disease, in some important points, I am induced to offer such members of the profession as are not already acquainted with the views of the French school upon this subject, an opportunity to form an idea of the same, leaving to them to select *e bonis optimum.*

Taking for granted that croup is generally, perhaps almost exclusively, met with in children, I am of the impression that we cannot look to any better and more proper source of information upon the subject, than to the "Hospital des Enfans Malades," in Paris, where Guersent, one of the most experienced practitioners in Paris, at the present time, and a physician to the above mentioned hospital, has been for many years observing the disease in children, of every age, and both sexes.

Contrary to the prevalent opinion in England, you will find that most continental writers have adopted Johnston's idea, that croup and angina maligna are of the same nature, which idea Bretonneau has more clearly brought into notice. He has, as Guersent says, demonstrated that the epidemic malignant angina is a genuine pellicular inflammation, similar to that of croup. He has also proved, that these two diseases, identical as to pathological changes, differ from each other only in the seat which they occupy. Angina maligna is therefore to be classed with Bretonneau's diphteritis, (from the Greek διφθερ, pellis, pellicula, exuvium,) by which Bretonneau understands one for the mucous membranes, and the skin in particular, which is very often an epidemical disease, in which the affected mucous surface is covered with a membraniform or pellicular exudation; more generally taking place in the mucous membranes of the fauces and the air passages, though also in mucous membranes of other organs. This doctrine of Bretonneau, Guersent has adopted in its full extent; so also has Laennec.

Before giving a more particular outline of Guersent's view of croup, I will attempt to illustrate what I have laid down by a diagram.

Diphteritis,

Croup, Angina maligna,

Croup (Pseudo-membranous,) or Laryngite caverneuse, Pseudo-croup, or Laryngite striduleuse.

Laryngitis stridulosa simplex, Laryngit. stridul. complicata.

Guersent entertains the impression that three different stages are to be observed in croup. In the commencement of the disease, in nineteen out of twenty patients, there are slight chills, with pain in the throat, and fever; and often a swelling of the submaxillary glands. The pharynx is reddened, and the tonsils are observed to be swelled, and to have upon their surface patches of a whitish concretion, (plaques,) which occupy either the tonsils, or velum palati, or uvula, or sometimes all these parts. This effusion Guersent has traced along all the passages which communicate with the fauces; we must not, therefore, be surprised to find diphteritical pseudo-membranes in other parts of the body. In this first stage may be recognised the true angina diphteritica. This period lasts from one to six or seven days.

In the second stage, our attention is attracted by a short and dry cough, occasionally returning, accompanied with aphony, and sometimes suffocation. While these symptoms increase, an alteration of the sound of the voice takes place, it becoming hoarse and dry. In genuine croup, (laryngite caverneuse,) the voice gradually loses its sonorousness, becomes feeble and very deep. The cough is hoarse, dry, and, so to say, suffocated. In the intermissions of the coughing, may be observed a kind of wheezing, which is so strong that it entirely prevents the natural vesicular sound of the lungs from being heard. The pain in the larynx or trachea occasioned by the cough, is greater than might be supposed. The respiration and pulse are frequent; the lips become livid; the face pale or livid, but during an exacerbation of fever, reddish; no convulsive movement or mental aberration is observed. Sometimes coughing produces an effusion of slimy or membranous matter, which for a moment eases the inspiration. The patient prefers to be silent, from the difficulty that speaking causes him. If the cough in this period takes a favourable course, it will decrease, and the patient will cough up a more or less transparent phlegm, mixed with small membranous pieces. Sometimes, however, the symptoms of the disease, after some days, return with a greater strength than ever. This increase of the symptoms forms the third stage of the disease, according to Guersent.

Still, these regular stages, Guersent observes, are not always seen in croup; it is sometimes found that the first and second stages make their appearance at the same time; sometimes the first stage is wholly absent. At other times, but very rarely, the disease commences in the bronchi, assuming an ascending character. This form, often mentioned as a mere possibility, Guersent has illustrated by a case, observed by him in the hospital, in which the pseudo-membrane ascended to the epiglottis, beginning at the bronchi.

As to the combination of croup with exanthematic disease, Guersent has only twice seen croup in scarlatina, and three times in small pox; nor has he found it oftener in measles, but he has observed a laryngitis stridulosa catarrhalis. Guersent further mentions a laryngitis variolosa, which he says may, by an inattentive observer, be mistaken for croup. In this affection, upon a post-mortem examination, are found upon the epiglottis, larynx, and even upon a part of the trachea, small round patches of a greyish blue colour, as if these parts were cauterized with muriatic acid.

I have thus attempted to present an idea of Guersent's views of true croup, which, in his opinion, is necessarily accompanied by a pseudo-membrane, and unless this pseudo-membrane be present, he calls all other forms of croup, as is seen from the diagram, pseudo-croup. This latter disease, pseudo-croup, it will be recollected, has two forms, being either simple or complicated. The former is far more common amongst children than the true croup. There may be observed two periods in this disease.

Generally, the disease is first observed in the evening or early in the night. The child starts suddenly from sleep, has a dry and hoarse cough, which is formed, as it appears, upon expiration; whereas, on the contrary, the cough, in genuine croup, is formed upon inspiration. From the sense of suffocation that takes place, and from the child's being suddenly awakened in this state, it is easy to account for the child's state of alarm. At the end of the attack the face turns pale instead of red, and there is a cold sweat. The voice is not altered; only a little hoarse: upon examination of the fauces, no pseudo-membrane, nor even swelling, is found. The pulse is more frequent than usual, but only under and immediately after the attacks; there is no fever.

The second form of the disease takes place two or three days after the appearance of a simple catarrh, and is to be treated accordingly. As Guersent has seen no death from this milder form, he has not had an opportunity for a post-mortem examination.

The last or complicated form may be combined with pneumonia, with a slight angina, and finally with nervous or adynamic affections. To the last class is to be referred the *nervous* croup; also, Millar's autumnal asthma, laryngismus stridulus of Mason Good. It is here proper to mention, that Guersent has, in children from five to twelve years of age, observed an asthma peculiar to this age, which is never met with in very young children; the disease is quite similar to the nervous asthma of adults.

Croup has a tendency to become epidemic both in hot and cold climates, according to circumstances; but we can with certainty assert that the disease is not contagious. Although the disease occurs in both sexes, males are much more disposed to it than females. Guersent tells us that pseudo-croup generally attacks children from one to seven years of age, and he has only twice found the disease in children beyond that age. It will be recollected that I stated that croup is almost exclusively met with in children: several eminent physicians have, indeed, reported cases of this disease in adults. Bretonneau states that he has seen a case occur at the age of sixty-two years. I have here presented an outline of the present views of the French schools with regard to croup. These views have met with much opposition, particularly in England.

We now come to the subject of the treatment of croup, and here there is not a little diversity of opinion. Much of this difference of opinion may be ascribed to the fact, that English and

American physicians are not usually called in before the disease has considerably advanced; whereas, in France, the physician is generally summoned in a much earlier stage of the disease.

It is generally admitted to be prudent to administer an emetic in the beginning of the disease. Dr. Meigs recommends the use of alum, a remedy already used by Bretonneau, by insiflation in the form of powder. Still, I should think we ought to be careful in administering it, as in large doses it would be apt to produce diarrhœa; Sachs has recommended it in combination with camphor, in angina tonsillaris. The next step in the treatment, according to the English writers, is the abstraction of blood, either generally or locally. According to them, a speedy and decided impression must be made upon the system by opening a vein, the bleeding to be pushed, according to some, *usque ad deliquium*. This treatment I should consider hazardous in children, particularly in infants. With respect to the abstraction of blood, which is not to be practised according to my view, before the second stage of the affection has appeared, Guersent says that he has never found venesection or leeching able to arrest, or even retard the course of the disease; but he does not therefore disapprove of it, but on the contrary, recommends it in plethoric subjects, as a means of gaining time to use other remedies. In laryngitis stridulosa complicata, venesection is certainly advisable, where pseudo-croup is complicated with pleuritis pneumonia, in which cases the venesection seems indicated rather by the accompanying disease than by pseudo-croup itself.

A more valuable and common remedy, with the French, is calomel. Although this drug is exhibited in croup, in England, and even recommended, we do not find the use of it carried to the same extent as in France, where, besides the internal use of it, Bretonneau and Guersent are in the habit of applying it externally, by the mode of inunction, not only round the neck, but also in the axillæ, and on the interior and upper part of the arm. They have observed good effects from salivation. As to the internal use of calomel, they particularly recommend small doses.

Another favourite practice in France, upon which Trousseau and Belloc depend very much, is the exhibition of caustic, which is to be applied only in Guersent's first stage of the disease. Bretonneau has highly recommended, for this purpose, muriatic acid, and Mackensie's nitrate of silver; Trousseau and Belloc go further than merely touching with caustic the mucous membrane,—they even sprinkle the interior of that organ with a syringe, constructed particularly for that purpose. Ryland recommends a combination of alum and honey, for this purpose. Finally, I must mention Autenreith's treatment of the disease, which consists exclusively in frictions of tartar emetic ointment, (lard ℥i., tart. emet. ʒiss.,) over the epigastrium. He found this method infallible in the epidemy in Tübingen: he did not lose a single patient, and reduced the course of the malady to as many days as it had previously run weeks. If we examine the results of these different modes of treatment, we must concede that there is a relative mortality in the whole, which is certainly owing to the greater attention which is of late paid to the disease. But if we ask, how it is possible for physicians, following about the same method in treatment, to report the different results that we read of? we can only account for this by referring to the different views entertained of the disease,—for instance, Guersent does not consider that there is any form of croup where there is not pseudo-membrane; but for pseudo-croup, Autenreith has probably not made this distinction—and we thus understand why he did not lose a single patient. As to the causes of death, it is generally said "that the child died suffocatively." But, although we of course must admit that there is always an encroachment upon the larynx, we cannot, on the other hand, but concede, that there is sufficient room for the passage of air to the lungs. I cannot, therefore, but assent to Guersent's opinion, that the final cause of death is cramp of the larynx and trachea—an opinion I am much strengthened in, from having seen in some cases great benefit derived from the application of the liquor ammoniæ fortis, and also of the use of the tobacco poultice, which I know one of the best physicians in this city is in the habit of applying.

When all other remedies fail, we must resort to tracheotomy, which, as it is now performed in France, in combination with the exhibition of topical remedies, is to be regarded of much greater use and influence than before. I leave to those of the profession, who have more experience in that department, to decide of its value and performance. Anceps remedium melius nullo.

